# Effect of Number and Location on Stress Distribution of Mini Dental Implant-Assisted Mandibular Kennedy Class I Removable Partial Denture: Three-Dimensional Finite Element Analysis

**DOI:** 10.1155/2022/4825177

**Published:** 2022-03-26

**Authors:** Wissanee Jia-mahasap, Chaiy Rungsiyakull, Wipahatpong Bumrungsiri, Natthaphorn Sirisereephap, Pimduen Rungsiyakull

**Affiliations:** ^1^Department of Prosthodontics, Faculty of Dentistry, Chiang Mai University, Chiang Mai, Thailand; ^2^Department of Mechanical Engineering, Faculty of Engineering, Chiang Mai University, Chiang Mai, Thailand; ^3^Roi Et Hospital, Roi Et, Thailand; ^4^Chiang Saen Hospital, Chiang Rai, Thailand

## Abstract

**Purpose:**

To investigate effects of number and location on patterns of von Mises stress distribution and volume average stress on abutment tooth, edentulous ridge, mini dental implant, and surrounding bone of mini dental implant-assisted mandibular Kennedy class I removable partial denture.

**Materials and Methods:**

Eight three-dimensional finite element models of mandibular Kennedy class I with different numbers and locations of mini dental implants were constructed. Mini dental implants were generated in the area of second premolar, first molar, and second molar, respectively. A static load of 400 N was applied on all models. The von Mises stress and volumetric average stress were calculated by three-dimensional finite element analysis.

**Result:**

The minimum volumetric average stress of abutment tooth was found in the model, where there was one mini dental implant at the second molar position and 2 mini dental implants at first molar and second molar positions. The model with three mini dental implants had reduced volumetric average stress of abutment tooth, which was not different from the model with two mini dental implants. However, the minimum volumetric average stress of mini dental implant and surrounding bone were found when three mini dental implants were applied, followed by two and one mini dental implants, respectively.

**Conclusion:**

Placing at least one mini dental implant at a second molar position can help reduce stress transferred to the abutment tooth. Stresses around each implant and surrounding bone reduced with increased numbers of mini dental implants.

## 1. Introduction

In the situation of partially edentulous patients, conventional clasp-retained removable partial denture (RPD) has been the treatment of choice for decades because of the noninvasive procedure and economically affordable treatment [1]. However, bilateral mandibular distal extension RPDs compared to maxillary distal extension RPDs and tooth-supported RPDs reveal limited anatomical supporting areas, which is susceptible to load transferred to underlying mucosa and residual ridge and may endanger the abutment teeth involved [2]. The use of dental implants as an ancillary component and implant-assisted removable partial dentures (IARPDs) has been encouraged by several authors [2–5]. IARPD can transform a Kennedy classification I and II into a Kennedy classification III by providing a posterior support to the prosthesis. [6, 7] Moreover, when the clasps of existing RPD provide insufficient retention or when the visibility of clasp is unaesthetic, the implants can be placed mesially adjacent to abutment teeth [8]. Cunha et al. [9] and Matsudate et al. [10] found that dental implant placed adjacent to abutment tooth help reduce occlusal force on abutment tooth better than dental implant placed distant from abutment tooth [9]. However, the retrospective study by Jensen et al. [11] reported higher technical failure rate of IARPD with support when implants were placed in the premolar position compared to second molar position.

The recent finite element analysis (FEA) by Messias et al. [5] showed that the dental implant placed in each of the bilateral edentulous areas can provide support and retention to the distal extension bases of the RPD. The vertical and anterior-posterior displacements were also reduced regardless of the implant positions. Furthermore, the meta-analysis by Park et al. [2] revealed that the IARPD in mandibular Kennedy class I partially edentulous patients promoted significant improvement in patient satisfaction and oral health-related quality of life (OHRQoL).

Several studies [6–8, 12–14] reported the use of standard-diameter implants with diameters ranging from 3.4–5.8 mm [15]. However, standard-diameter dental implants still have some limitations for several patients such as geriatric patients or patients with insufficient bone architecture. Therefore, there has been an attempt to use mini dental implants, a dental implant that is fabricated with a reduced diameter (less than 3.0 mm) and shorter in length with the same biocompatible material as compared with standard dental implants [16–22]. Omran et al. [17] reviewed that when mini dental implants were used with complete denture, the survival rate was 95–97%. However, Bourauel et al. found that the bone implant contact (BIC) between 2.8 mm diameter dental implant and the surrounding bone has 37% lesser contact area than the BIC of the 3.8 mm diameter dental implant [23, 24]. Moreover, implant diameter was more critical for improved stress distribution than implant length. A greater implant diameter help reduced the stress around the implant [25–27]. In small diameter implants, stress distribution increased in the surrounding bone compared to larger diameter implant in both vertical and lateral loads. [28] Therefore, reports indicate the need to increase number of mini dental implants when used with complete denture [15, 29]. However, there was still scarce information which described appropriate number of mini dental implants when used as IARPDs. Up to date, the placement of small diameter dental implants on distal extension ridges helps to reduce the stress concentration on denture supporting structures, and the maximum von Mises stress is affected by the different designs of clasp components [30].

This study aims to investigate the pattern of stress distribution at abutment tooth, mini dental implant, and surrounding bone using various models with different numbers and locations of mini dental implants.

## 2. Material and Methods

### 2.1. Construction of Mini Dental Implant-Assisted Mandibular Kennedy Class I Removable Partial Denture Model

Mandibular Kennedy class I bilateral distal extension model was constructed to simulate edentulous area 35–37 and 45–47 using self-polymerizing acrylic (Vertex Self Curing, Soesterberg, the Netherlands), representing the hard tissue. The model was covered with 2 mm thick silicone gingival tissue to simulate the soft human tissue. The ranges of Shore hardness A silicone material (14 to 16) are similar to those of the human soft tissue (16 to 21) [31]. The acrylic resin teeth 34–44 (PE-ANA002®; Nissin, Kyoto, Japan) were installed in the model with an artificial periodontal ligament, simulated with silicone impression material (GI Mask). The thickness of artificial PDLs was 0.3 mm [32]. The conventional cobalt-chrome-molybdenum RPD framework, with lingual bar and rest-proximal plate-Akers (RPA) clasp engaged mesial undercut of both first premolar abutment teeth, was fabricated in the model [33].

The mini dental implant diameter 2.7 × 10 mm (PW PLUS CO., LTD., Nakorn Pratom, Thailand) was used for IARPD. Equator attachment (OT Equator®, Rhein83, Italy) and its corresponding retentive cap (Retentive cap, Rhein83, Italy) were attached to the implant.

### 2.2. Transfer of the Model into a Digital File

The 3Shape lab scanner (3Shape lab scanner D850 Copyright© 3Shape A/S., Denmark) and intraoral scanner (TRIOS® intraoral scanner Copyright© 3Shape A/S., Denmark) were used to scan the mandibular model, RPD framework, and mini dental implant. The scanned data was transferred into digital file in the DICOM file format then converted to STL file by the TRIOS® Orthodontics software.

**Figure 1:**
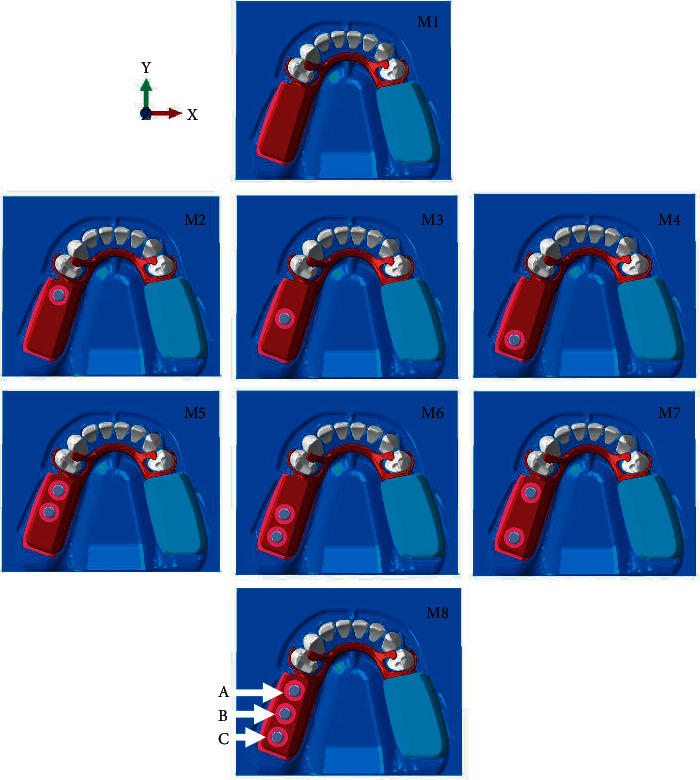
Eight models classified by locations and numbers of mini dental implant. Implant at second premolar (A), first molar (B), and second molar (C) were distally placed 6.5, 11.5, and 16.5 mm from abutment tooth, respectively.

Solid Works Software, version 2015 (Software Solid Works, version 2015, Dassault System, France) was used to generate the mandibular model, RPD framework, and mini dental implant with equator attachment. The data were recorded in SLDPRT file. Eight models of mini dental implant-assisted mandibular Kennedy class I bilateral distal extension RPD were generated by 6.13 ABAQUS program (ABAQUS 6.13, Simulia, Providence, RI, USA) with different numbers and locations of mini dental implants (Figure 1). Mini dental implants were placed at second premolar, first molar, and second molar position distally from abutment tooth 6.5, 11.5, and 16.5 mm, respectively [33]. The measurement from a strain gauge model under the same conditions was utilized to validate the 3D FEA model at the surface area of FE model. The results obtained from both FEA and strain gauge measurements were correlated within an error margin of less of 10%, which was an average of the gauges obtained.

### 2.3. Three-Dimensional Finite Element Analysis

The relationship of each element was specified to enhance mesh with tetrahedral element type C3D4 with 4 nodes, while some locations with acute angle, tetrahedral elements type C3D10 with 10 nodes, hexahedral elements type C3D8R with 8 nodes, and wedge elements of type C3D6 with 6 nodes were applied. Each model, therefore, had a different numbers of nodes and elements as shown in Table 1.

The elastic modulus and the Poisson ratio for each material are summarized in Table 2 [34–38], in which the same material properties are specified for both cementum and tooth dentin due to similar mechanical properties. The average Young's modulus of bone was calculated as follows [37]:

(1) $$\begin{array}{c} {\text{E}}_{\text{Avg}}=\frac{((Vc\times Ec)+(Vt\times Et))}{\left(Vc+Vt\right)}. \end{array}$$

E_Avg_ was the average Young's modulus of both types of bone. *Vc* represented the volume of the cortical bone, while *Ec* represented Young's modulus of the cortical bone. *Vt* represented the volume of the trabecular bone, while *Et* represented Young's modulus of the trabecular bone. Young's modulus of cortical bone and trabecular bone were 14700 and 490 MPa, respectively, with the same Poisson's ratio of 0.30 for both types of bone.

**Figure 2:**
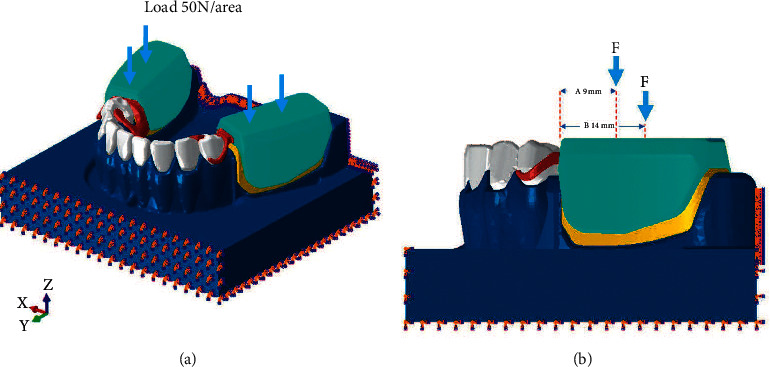
Full isotropic view of the FE model with loading and boundary condition (a) displays model capture at the front part (b) displays the loading location at the side view of the model with location of loading 9 and 14 mm far from the abutment.

All materials were assumed to be linearly elastic, homogenous, and isotropic to simplify the calculations. The nodes of the most medials and most distal surfaces in the models were constrained in all directions. Tie contacts were applied to all surfaces, except the surface between clasps and abutment teeth, which was set as a frictional contact with the friction coefficient of 1. The vertical load of 100 N [39] was applied bilaterally on the acrylic distal extension denture base at 9 and 14 mm distal from first premolar abutment tooth (Figure 2). The models were given a 10% mesh convergence test to confirm that the results of this study would not be interfere by mesh amount. The volume average of von Mises stress was calculated on the abutment tooth, mini dental implant and surrounding bone [40]:

(2) $$\begin{array}{c} \text{Volume average of von Mises stress}=\frac{\Sigma \left(\text{stress}\times \text{volumebyelement}\right)}{\Sigma \left(\text{volume}\right)}. \end{array}$$

## 3. Result

**Figure 3:**
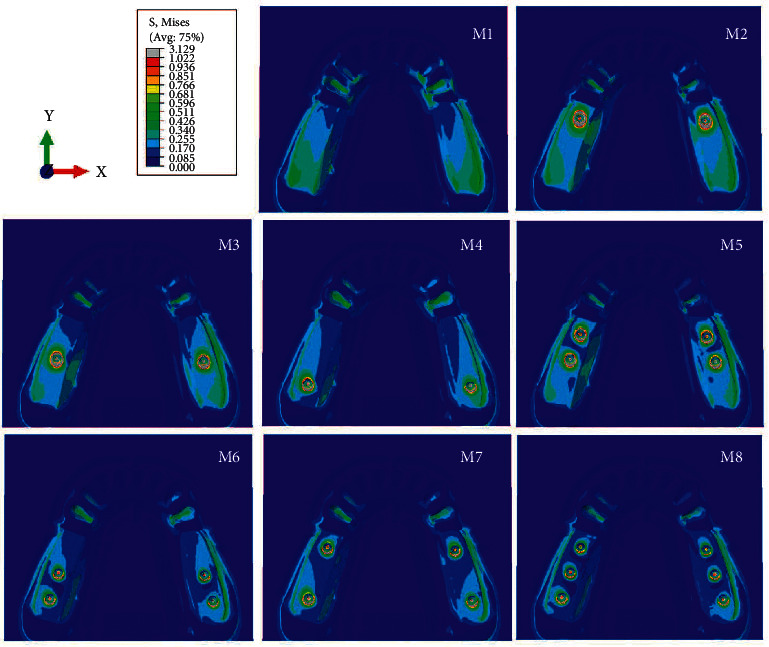
Stress pattern occurring in model nos. 1–8.

**Figure 4:**
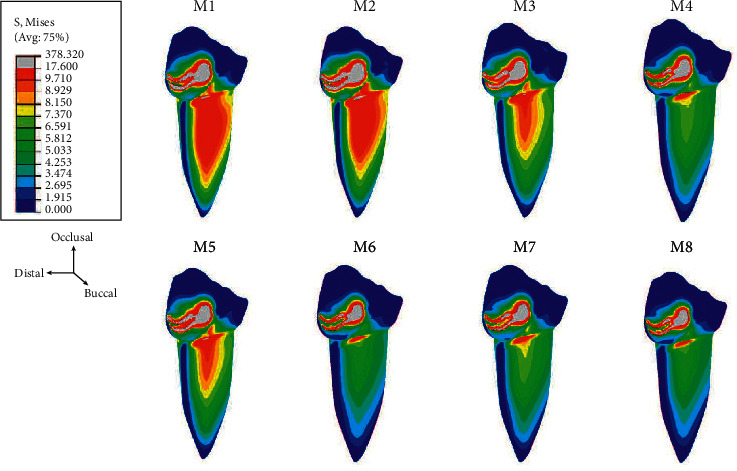
Stress distribution on the distal surface of abutment tooth's root.

When comparing 8 models, in model 1 (M1) without mini dental implants, the average volume of von Mises stress was concentrated at the cervical part of the abutment tooth and distal extension edentulous area, whereas the stress was greatest at second molar edentulous area and reduced anteriorly. Contrarily, in model 2 to 8 (M2–M8), with mini dental implants, the stress was reduced at edentulous area and concentrated more at the abutment tooth and mini dental implant (Figure 3 and Table 3) when there was at least one mini dental implant present at the second molar position (M4, M6, M7, and M8). The average volume of von Mises stress at the abutment tooth was reduced (Figure 4).

**Figure 5:**
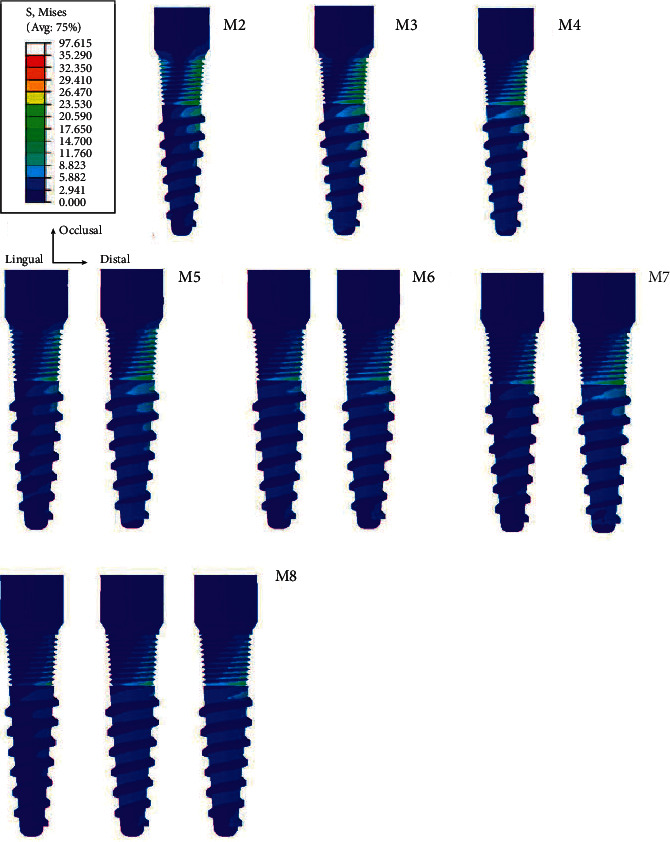
Stress distribution on the missal surface of the dental implant.

**Figure 6:**
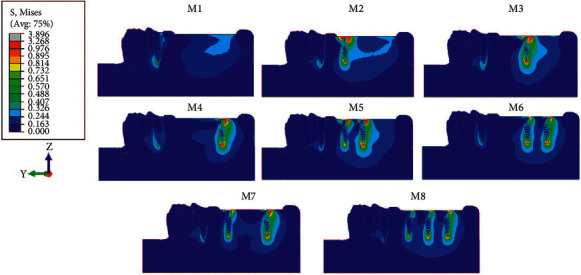
Stress distribution at the surrounding bone of the dental implant.

Regarding the numbers of mini dental implants, when three implants were placed, the stresses accumulated at each implant reduced when compared to using two and one implants, respectively. Furthermore, the stress at each implant in all models was concentrated on the distal side in the coronal 1/3 area of the implant (Figure 5). Considering the average volume of von Mises stress in the surrounding bone, the stress in the peri-implant region was found concentrated at the neck of the mini dental implant on the distal side and around the apex of the implant and was greater than in regions distant from the implant (Figure 6).

## 4. Discussion

With regard to implant location, it was observed that distally placed implants resulted in reduced stress at the edentulous area because it transformed mandibular Kennedy class I to a more favorable arch configuration: mandibular Kennedy class III. Kennedy class III configuration's eliminated lever arm usually occurred with distal extension RPD, which reduced distal displacement of the soft tissue. Furthermore, load transfer to the abutment teeth was also reduced [41, 42]. This is in agreement with the systematic review of Zancope et al. [7] which explained that implant placement at the most posterior region for IARPD provided optimal stability for the prosthesis. Similarly, Grossmann et al. [6] recommended placing implants at a second molar position to enhance support and stability. A retrospective study by Mitrani et al. [43] also revealed that implant placed posteriorly from the fulcrum line helped improve support and retention for distal extension RPD because it prevents tissue intrusion from the denture base and rotation under masticatory force. Furthermore, implant placed distally also increased patient satisfaction and masticatory performance without impairing implant survival.

In contrary, Memari et al. [42] found the lowest stress on the residual ridge when the implant was placed in the first molar region and the highest stress was observed when the implant was placed in the second molar area. Cunha et al. [9] reported contradicting results. The displacement of soft tissue at the distal extension area reduced when the implant was placed adjacent to the abutment tooth. Furthermore, load transfer to the abutment tooth also reduced when the implant was placed approximating the abutment tooth. A recent study by Messias et al. [5] encouraged placing one implant in each of bilateral distal extension areas regardless of the position, as this can minimize the denture base displacement vertically and anteroposteriorly, which reduces abutment tooth mobility.

**Figure 7:**
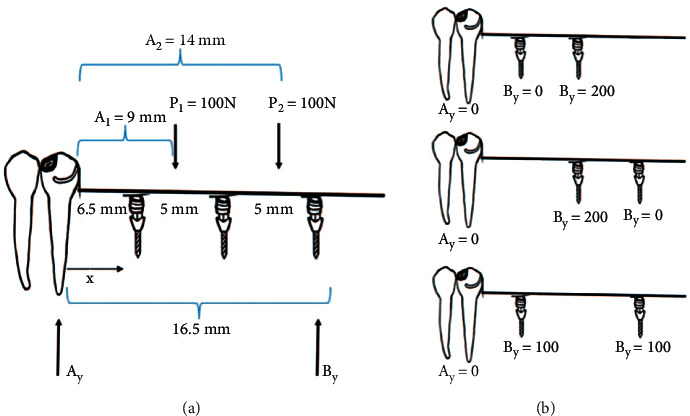
Diagram of the bending moment calculation. (a) represents action force and the distance between the abutment tooth and dental implant. A_y_ denotes the action force on the abutment tooth. B_y_ denotes the action force on the dental implant. (b) Diagram of loading on abutment and dental implant with 2 assisted dental implants on removable partial denture.

In terms of implant number, using one or two mini dental implants that were not placed at the far distal position revealed more load transfer to abutment teeth when compared to one mini dental implant placed distally. It was assumed that even though there were more implants supporting the denture base, there was still distal lever arm which allowed denture base displacement, causing torque to abutment teeth. However, when three mini dental implants were placed, load transfer to abutment teeth was not different when compared to having one or two mini dental implants placed distally at the second molar position. This can be explained via bending moment loading calculation as per equations (3)–(6) and Figure 7 [44].

(3) $$\begin{array}{c} \sum F=0,\;P-V=0, \end{array}$$

(4) $$\begin{array}{c} \sum M=0,\;M-Px=0, \end{array}$$

where *F* = total force/resultant force, *P* is the action force, *V* is the shear force, *M* is the bending moment, and *x* is the distance from the abutment tooth to the location used in calculating shear force and bending moment.

Distance 1 is given by [44]

(5) $$\begin{array}{c} V=Ay,\\ M=Ayx,\left(0<x<a1\right). \end{array}$$

Distance 2 is given by [44]

(6) $$\begin{array}{c} V=Ay-P1,\;M=Ayx-P1\left(x-a1\right)\left(a1<x<a2\right). \end{array}$$

Referring to Figure 7 and equations (3)–(6), when two mini dental implants were placed, the load on the abutment tooth was equal to zero with load distributed to both implants instead. Therefore, increasing the number of dental implants to three implants did not help reduce stress on abutment tooth. However, placing three mini dental implants still have some benefit as it helped decrease stresses applied on each implant and surrounding bone.

A study by Messias et al. [5] showed different patterns of vertical displacement of prosthesis in distal extension RPD when there were no implants, an implant placed approximate the abutment tooth, and even with an implant placed distant from the abutment tooth. There was still occlusal load distributed along the edentulous length where the teeth were replaced. Therefore, concordance with our study that when additional support is needed in the distal extension edentulous area, more implant installation would help distribute load to the implants and reduce soft tissue displacement.

The clinical implication obtained from the 3D FEA was that the mini dental implant can be used to assist the mandibular Kennedy class I RPD. When there was at least one implant placed distally at the second molar position, it can minimize the stress emerging at the abutment tooth. Furthermore, in the situation when patients have heavy bite forces, such as patients with parafunctional habits, long-span edentulous ridges, and natural dentitions present in the opposing arch, increasing the number of mini dental implants to two or three implants would help distribute load applied on each implant.

However, the limitation of this study was that only the vertical load was applied to the distal extension area. Consequently, it may not clinically represent load distribution from lateral movement of the denture base at the edentulous area. Another limitation of this work was that in FEA, all materials were assumed to be linearly elastic, homogenous, and isotropic; however, this may not clinically represent the occurring situation. The RPDs were not rigidly bonded to the associated structure intraorally. Therefore, the load transfer may vary in the connection area, for example, the connection between equator abutment and the implant platform. Further study is needed to evaluate the optimal number of mini dental implants required in different edentulous spans and different biting forces.

## 5. Conclusion

Within the limitation of this study, the following conclusions were drawn.

1. Placing at least one mini dental implant at a second molar position can help reduce stress transferred to abutment teeth.
2. Using two and three mini dental implants can help reduce stress around each implant and surrounding bone when more support to the prosthesis is necessary.

## Figures and Tables

**Table 1:** Properties of finite element model components.

Model	Node	Element
1	550036	880152
2	670179	1348329
3	669352	134857
4	669263	1340328
5	785416	1812039
6	785134	1809050
7	781410	1799889
8	901181	2279392

**Table 2:** Material properties of finite element models [34–38].

Material	Young's modulus (MPa)	Poisson's ratio
Tooth dentin	18600	0.31
Bone	4042.5	0.30
Acrylic resin	2200	0.31
Cobalt chromium metal	211000	0.30
Titanium dental implant	110000	0.33
Periodontium	68.90	0.45
Connective tissue	0.68	0.45
Stainless steel	19000	0.31
Rubber cap	5	0.45

**Table 3:** Average volume stress of eight models.

Model	Dental implant location	Average volume stress (MPa)		
		Abutment tooth	Dental implant	Surrounding bone
1	None	3.31	-	-
2	Second premolar	3.46	4.72	0.40
3	First molar	2.75	4.87	0.45
4	Second molar	2.04	4.39	0.43
5	Second premolar, first molar	2.88	3.72	0.36
6	First molar, second molar	1.76	3.33	0.34
7	Second premolar, second molar	2.12	3.38	0.33
8	Second premolar, first molar, and second molar	1.83	2.81	0.29

## Data Availability

The data used to support the findings of this study are available from the corresponding author upon request.
